# Efficacy and safety of magnetic resonance-guided focused ultrasound for the treatment of painful bone metastases: a systematic review and meta-analysis

**DOI:** 10.1007/s00256-021-03822-8

**Published:** 2021-05-21

**Authors:** Joe D. Baal, William C. Chen, Ulysis Baal, Sagar Wagle, Jed H. Baal, Thomas M. Link, Matthew D. Bucknor

**Affiliations:** 1 Department of Radiology and Biomedical Imaging, University of California, San Francisco, 185 Berry Street, Lobby 6, Suite 350, San Francisco, CA 94107 USA; 2 Department of Radiation Oncology, University of California, San Francisco, San Francisco, USA; 3 Department of Radiology, Mayo Clinic, Rochester, USA

**Keywords:** MRgFUS, Bone metastases, Palliative, Efficacy, Safety, Meta-analysis

## Abstract

**Objective:**

To report the safety and efficacy of magnetic resonance-guided focused ultrasound (MRgFUS) in the treatment of painful bone metastases through a systematic review and meta-analysis of pain scores before and after MRgFUS treatment and post-treatment adverse events.

**Materials and methods:**

A comprehensive literature search of PubMed and Embase databases was performed for studies evaluating the efficacy and/or safety of MRgFUS. The mean difference of pain scores (10-point visual analogue scale or numerical rating scale) between baseline and 1-month/3-month pain scores was collected and analyzed in a pooled meta-analysis. Post-treatment adverse events based on the Common Terminology Criteria for Adverse Events (CTCAE) grading were recorded and the pooled prevalence was calculated.

**Results:**

A total of 33 studies published between 2007 and 2019 were collected, resulting in a total sample size of 1082 patients. The majority of the studies were prospective with a reported follow-up period of 3 months. The pooled proportion of patients that achieved pain relief from MRgFUS (complete response or partial response [≥ 2-point improvement of pain score]) was 79% (95% CI 73–83%). The pooled 1-month and 3-month mean difference in pain score were − 3.8 (95% CI − 4.3; − 3.3) and − 4.4 (95% CI − 5.0; − 3.7), respectively. The overall rate of high-grade (CTCAE grade 3 or higher) and low-grade (CTCAE grade 2 or lower) MRgFUS-related adverse events were 0.9% and 5.9%, respectively.

**Conclusion:**

MRgFUS is an effective procedure that is able to provide significant pain palliation for patients with symptomatic bone metastases with a favorable safety profile.

## Introduction

With the recent advances in preventive measures and cancer treatment, there has been a yearly decline in cancer mortality rates worldwide [1, 2]. This has led to an increasing number of patients living with advanced cancer, a portion of which carry debilitating complications. After the lungs and the liver, bone is the third most frequent site for metastasis in advanced cancer [3]. Bone metastases can indicate short-term prognosis, but are often also a major cause for morbidity, typically characterized as severe pain that can impair mobility and overall quality of life [4]. With the median survival of patients with bone metastases ranging 0.5 to 4 years, successful palliative treatment of these painful bone metastases is important for preserving quality of life improvement [5].

Currently, the standard local palliative treatment for patients with osseous metastatic disease is external beam radiotherapy. However, local radiotherapy for painful bone metastases has been associated with delayed side effects [6, 7]. Additionally, 30–40% of patients will not have significant improvement in pain [8, 9]. Re-irradiation of recurrent painful bone metastases is an available option but also increases the risk of radiation-induced adverse effects like pathologic fractures and myelopathy. As such, re-irradiation therapy is limited by radiation dose limits of the bone and its surrounding tissue [10–12].

In recent years, magnetic resonance-guided focused ultrasound (MRgFUS) has emerged as a safe and effective thermal ablation technique in the palliative treatment of painful bone metastases, and has been evaluated in a number of prospective studies and small clinical trials [13–45]. Published cohort studies on the use of MRgFUS for painful bone metastases have been limited in sample size which limits estimation of palliative treatment efficacy and safety.

To the best of our knowledge, a systematic review and meta-analysis on the treatment efficacy and associated toxicity of MRgFUS for painful bone metastases has not been performed. Therefore, the purpose of this study was to classify and pool selected literature to better define the efficacy and safety of MRgFUS for the palliative treatment of painful bone metastases.

## Materials and methods

### Literature search

A systematic search was conducted in March 2020 using the PubMed and Embase electronic databases with the query (“mrgfus” or “hifu” or “focused ultrasound”) AND (“bone” or “bone metastases” or “bone metastasis”). No time limitation was imposed on the search criteria. The list of potential studies was screened for relevance based on the titles and abstracts. References of selected studies were examined for other relevant articles. Non-English and duplicate studies were removed. Literature search and initial study screening were performed by a single investigator (JDB).

### Inclusion and exclusion criteria

Studies reporting clinical outcome and/or toxicity data of MRgFUS of painful bone metastases in five or more patients were included in this meta-analysis and systematic review. Studies were excluded if (1) there was no toxicity or outcome data (proportion of patients that respond to treatment and/or pain scores from baseline and follow-up) specific to MRgFUS treatment of painful bone metastases; (2) the follow-up period was less than 1 month; (3) the study reported redundant patient cohorts already reported in another study; (4) the study was a review, commentary, or editorial; or (5) the study had less than 5 patients. Preferred Reporting Items for Systematic Reviews and Meta-Analyses (PRISMA) checklists were utilized for the study selection process [46].

### Data extraction

Further review and data extraction were performed by four investigators (WCC, UB, JHB, and SW) in all studies that met inclusion and exclusion criteria. Subsequently, a single investigator (JDB) reviewed the final data set from all included studies, and adjudicated any discrepancies with the other investigators to reach a consensus. The recorded data included the study (author name and publication year), number of patients, sex, median/mean age, median follow-up duration, proportion of primary malignancy (lung, prostate, breast, renal, colorectal, and other/unknown primary cancer), location of metastases (pelvis, extremity, ribs, spine, scapula), mean and standard deviation of pre-treatment pain score (10-point numerical rating scale [NRS] or visual analogue scale [VAS]), mean and standard deviation of post-treatment NRS or VAS scores at 1 month and 3 months, and number of patients achieving treatment response. Treatment response was a combination of complete response (pain score of 0 after treatment) and partial response, which was defined as ≥ 2-point reduction in the pain score, per the updated international consensus on palliative radiotherapy endpoints for future clinical trials in bone metastases [9]. A list of post-treatment adverse effects was recorded from each study and subsequently graded by severity based on the Common Terminology Criteria for Adverse Events (CTCAE), if not already graded [47]. For studies with available data, pain medication intake and quality of life scores before and after MRgFUS treatment were also collected.

### Statistical analysis

A pooled analysis was performed on the study weighted proportion of patients who experienced pain relief following MRgFUS treatment. Both fixed effects and random-effects models were applied. Hedges’ *g* statistic was calculated to quantify the change from baseline pain score (NRS or VAS) and follow-up pain scores. For some studies where it was not reported, pain score standard deviation was estimated from the 95% confidence interval and sample size using this formula: $\sqrt{N} \times (\left(\mathrm{upper CI}-\mathrm{Lower CI}\right) / (t \times$ 2)), where *N* is the sample size and *t* is the *t*-statistic [48]. The proportion of severe toxicity (CTCAE grade 3 or higher) was reported as an overall number as the reported rates were frequently zero and consistently low. Meta-analysis of binomial proportions was performed with the *metaprop* function in the *meta* package (v4.9–6) of R language (v3.6.1) [49].

## Results

### Study characteristics

**Fig. 1:**
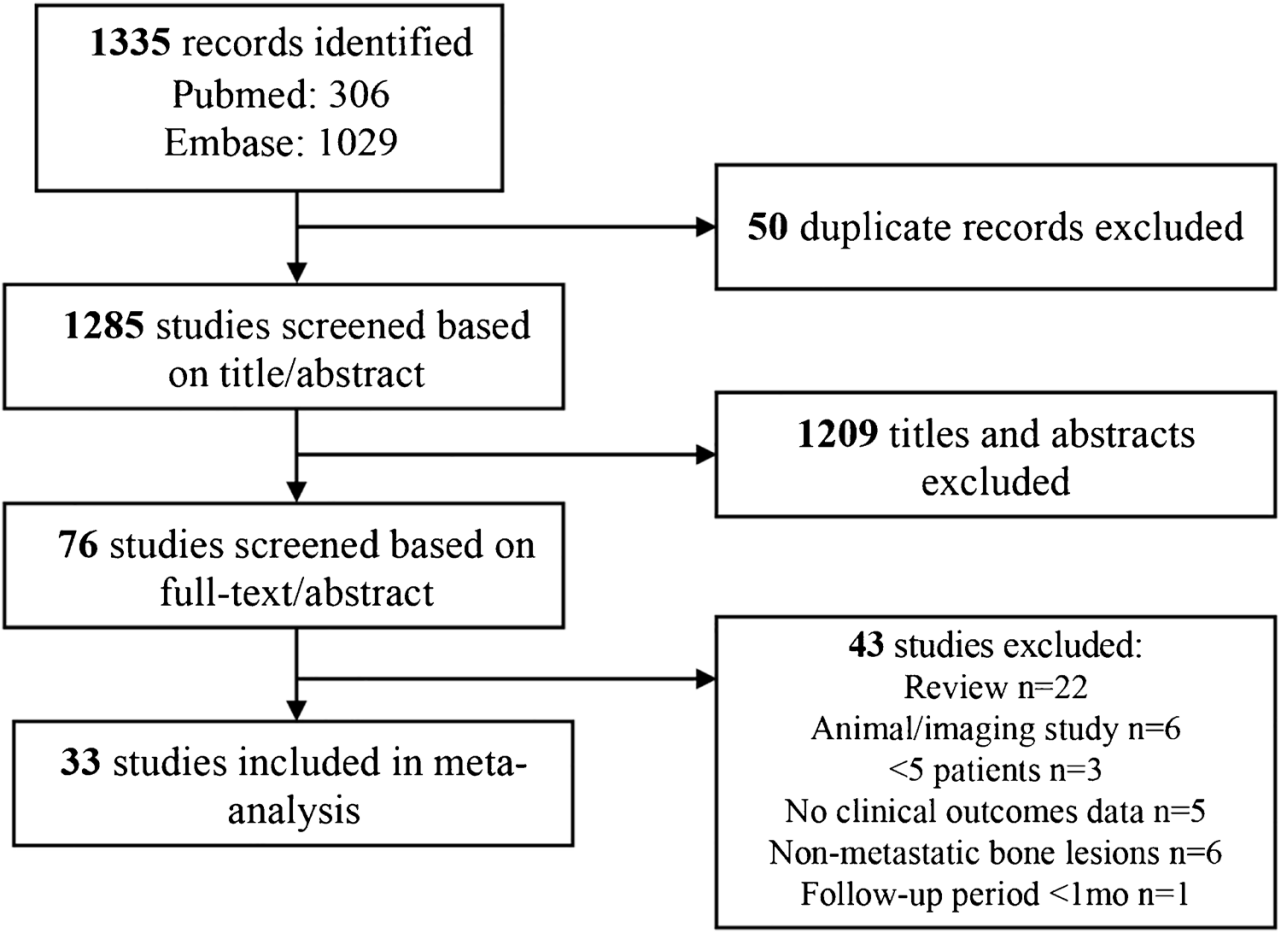
Flowchart of literature search and study selection process

A total of 1335 studies were identified through a literature search in PubMed and Embase (Fig. 1). Ultimately, 33 studies that met inclusion and exclusion criteria were selected for the meta-analysis [13–45]. The selected 33 studies were published between 2007 and 2019 and comprised three randomized control trials, six retrospective studies, and 24 prospective studies. Of note, 13 studies were in abstract-only format. Overall, the studies resulted in a total of 1082 patients with painful bone metastases treated with MRgFUS. Of the included studies, 26 reported data on MRgFUS-related adverse events (*N* = 799), 20 reported baseline and follow-up pain scores (*N* = 543), and 20 reported the overall proportion of patients that were able to achieve treatment response (*N* = 636). The median study sample size was 21 patients (range 5–140) with a median follow-up period of 3 months (29 studies, range 1–12). The median age of patients was 60 years (22 studies including one study on a pediatric study population, range 4.3–69). Twenty studies outlined primary malignancy type, which were mostly breast (30.2%) followed by lung (14.3%), renal (14.0%), and prostate (10.0%). Twenty-one studies provided data on bone treatment site, which were predominantly in the pelvis (72.2%), followed by the ribs (14.1%), extremities (13.9%), scapula (4.3%), and spine (1.2%). Table 1 provides summary study characteristics and Table 2 outlines study level specifics.

**Table 1:** Characteristics of pooled patient population

Number of studies	33 (13 abstracts)
Number of patients	1082 (median 21, range 5–140)
Median age (22 studies)	60.0 (range 4.3–69.0)
Median follow-up (months; 29 studies)	3.0 (range 1.0–12.0)
Primary malignancy (20 studies, *N* = 470)	
Prostate (*N* (%))	47 (10.0)
Breast	142 (30.2)
Lung	67 (14.3)
Renal	66 (14.0)
Colorectal	20 (4.3)
Liver	7 (1.5)
Other	121 (25.7)
Bone location (21 studies, *N* = 575)	
Pelvis (*N* (%))	415 (72.2)
Ribs	81 (14.1)
Extremities	80 (13.9)
Scapula	25 (4.3)
Spine	7 (1.2)
Overall treatment response (20 studies, *N* = 636)	502 (78.9)
Median pain scores (20 studies, *N* = 543)	
Baseline	6.7 (range 3.8–8.0)
1 month after treatment	3.0 (range 1.3–5.3)
3 months after treatment	1.9 (range 0.5–4.8)
Adverse events (26 studies, *N* = 799)	
High-grade toxicity (grade 3 +)	7 (0.9%)
Low-grade toxicity (grades 1–2)	47 (5.9%)

**Table 2:** Study-specific characteristics

Author, year	N Pts	Age	F/u (mo)	N TR (%)	Baseline PS	Baseline SD	1mo PS	3mo PS	3mo SD	N ≥ G3 tox (%)	N < G3 tox (%)	FUS system
Namba, 2019 [13]	10	69	3	8 (80)	6	2.7	3	2	4.1^*^			InSightec
Tsai, 2019 [14]	31	60	3	26 (84)	5.6	1.8				0 (0)	4 (12.9)	InSightec
Wang B., 2019 [15]	30	4.3	3	26 (87)	6.6	2.4	3.4	1.8	0.96	0 (0)	8 (2.7)	InSightec
Giles, 2019 [16]	9	52.6	3	6 (67)	7.3	1.4	2.5	1.5	2.2	0 (0)	2 (2.2)	Profound Medical
Dababou, 2019 [42]	140		12	109 (78)								N/A
Marrocchio, 2019 [43]	11									0 (0)	0 (0)	N/A
Bertrand, 2018 [17]	17	61	1	14 (82)	7.5	1.33	1.9					InSightec
Wang S., 2018 [18]	21		3		7.7	1.6	3.9	3.7	2.7			N/A
Chen Z., 2018 [19]	26	60	12		6.7	1.5	4	4.8	1.1			InSightec
Harding, 2018 [20]	18	57	3	13 (72)	7	1				0 (0)	0 (0)	Philips
Xiong, 2018 [22]	14		3		6.5	4	5.25	2.5	4.75	1 (7.1)	5 (3.6)	N/A
Lee H., 2017 [21]	21	59	3	16 (76)	6.57	1.6	2	1	1.4^*^	0 (0)	2 (9.5)	InSightec
Bazzocchi, 2017	11	56	3		3.8	2.8		1.8	2.3	0 (0)	0 (0)	N/A
Chan M., 2017 [24]	6	68.5	1	6 (100)			2			0 (0)	0 (0)	Philips
Anzidei, 2016 [25]	23	63.6	6	22 (96)	7.09	1.8	2.65	1.04	1.91	0 (0)	0 (0)	InSightec
Wang Q., 2016 [26]	71		3		6.7	1.82	4	3.7	2.81			N/A
Gu, 2015 [27]	23				6	1.5	3.1	2.2	1	0 (0)	4 (17.4)	InSightec
Joo, 2015 [28]	5	60	12		5.92	1.28	2.92	0.75	1	0 (0)	1 (20.0)	InSightec
Chen L., 2014 [29]	7		3		8	1.1	3	3.4	1.5	0 (0)	0 (0)	N/A
Huisman, 2014 [30]	11	60	1	7 (64)	7.54	3.75	3.82			0 (0)	2 (18.2)	Philips
Hurwitz, 2014 [31]	112	61.7	3	72 (64)	7	3.1	3.3	3.3	3.4^*^	3 (2.7)	11 (9.8)	InSightec
Pfeffer, 2014 [32]	22	69	3	16 (73)						0 (0)	0 (0)	N/A
Turkevich, 2014 [33]	32	57	3	32 (100)	6.8		1.9	1.1		0 (0)	0 (0)	InSightec
Zaccagna, 2014	72	62	6	63 (88)	6	6.3		2	6.3^*^	0 (0)	0 (0)	InSightec
Boni, 2013 [44]	11			8 (73)								N/A
Napoli, 2013 [35]	18	63	3	16 (89)	7.1	2.08	2.5	1	1.1	0 (0)	0 (0)	InSightec
Pfeffer, 2013	17	59	3	13 (76)	7.6		3.1	2.7		0 (0)	0 (0)	N/A
Noce, 2013 [45]	36	60.3	3							0 (0)	0 (0)	InSightec
Meyer, 2013	115									3 (2.6)	1 (0.9)	N/A
Catane, 2012 [38]	93		3		6.9		3.3	2.6		0 (0)	6 (6.5)	N/A
Liberman, 2009 [39]	25	61	3	18 (72)	5.9	0.8	2.3	1.8	1.4^*^	0 (0)	0 (0)	InSightec
Gianfelice, 2008 [40]	11	59	3	11 (100)	6.27	1.73	1.3	0.5	0.54	0 (0)	0 (0)	InSightec
Catane, 2007 [41]	13		6		5.5		1.7	1.2		0 (0)	1 (7.7)	InSightec

^*^Estimated SD

*PS*, pain score, *TR, treatment response*

### Treatment response

**Fig. 2:**
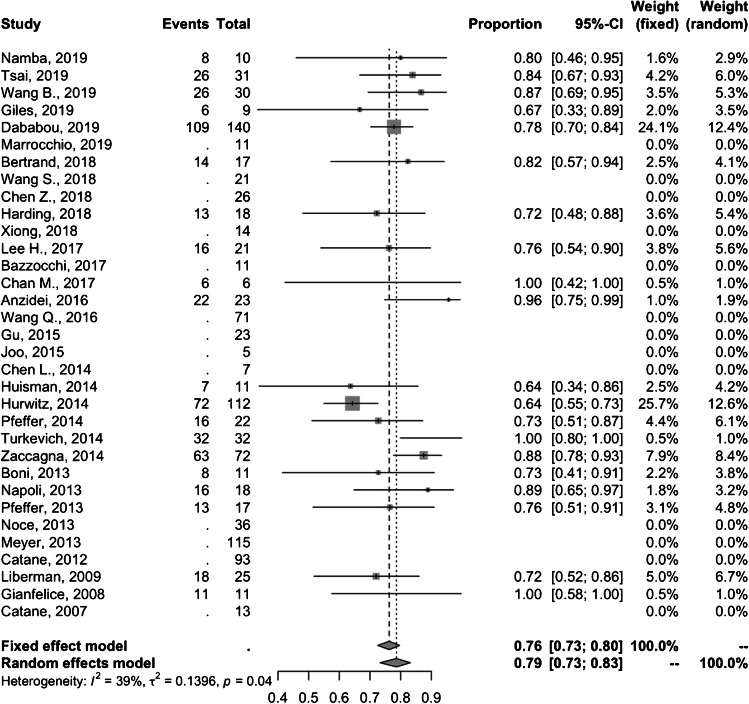
Forest plot for overall treatment response following MRgFUS treatment of painful bone metastases. Each study is identified by first author and year of publication, with the box representing individual study point estimates, the size of the box indicating the relative contribution to pooled estimate, horizontal lines signifying 95% CIs, and diamonds marking the fixed and random-effects pooled estimates

In 20 studies (*N* = 636), 502 (78.9%) patients were reported to have treatment response. Random-effects pooled proportion of overall treatment response was 79% (95% CI 73–83%) (Fig. 2). Out of the 502 treatment responders, 295 were further classified into groups achieving complete and partial response to treatment; 149 (50.5%) had complete response and 146 (49.5%) achieved partial response.

**Fig. 3:**
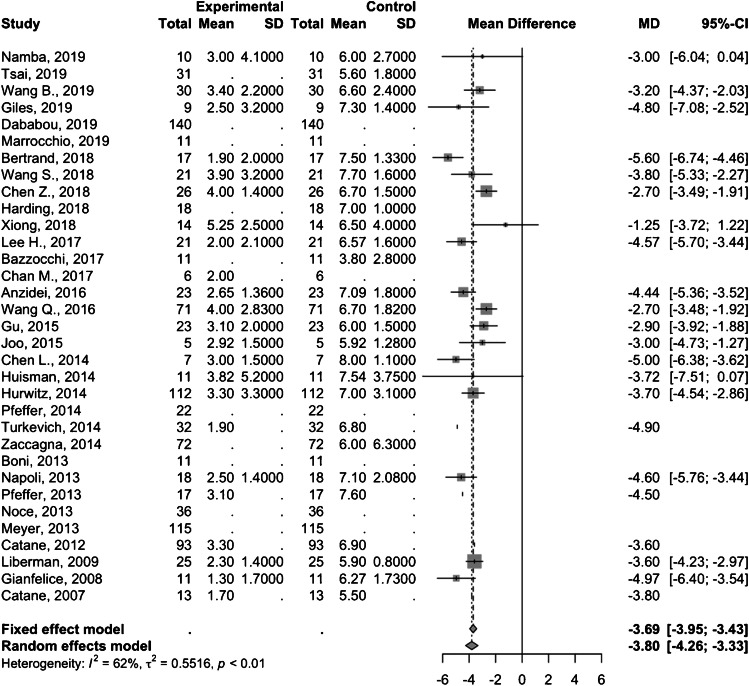
Forest plot of the mean difference in pain scores 1 month after MRgFUS treatment

**Fig. 4:**
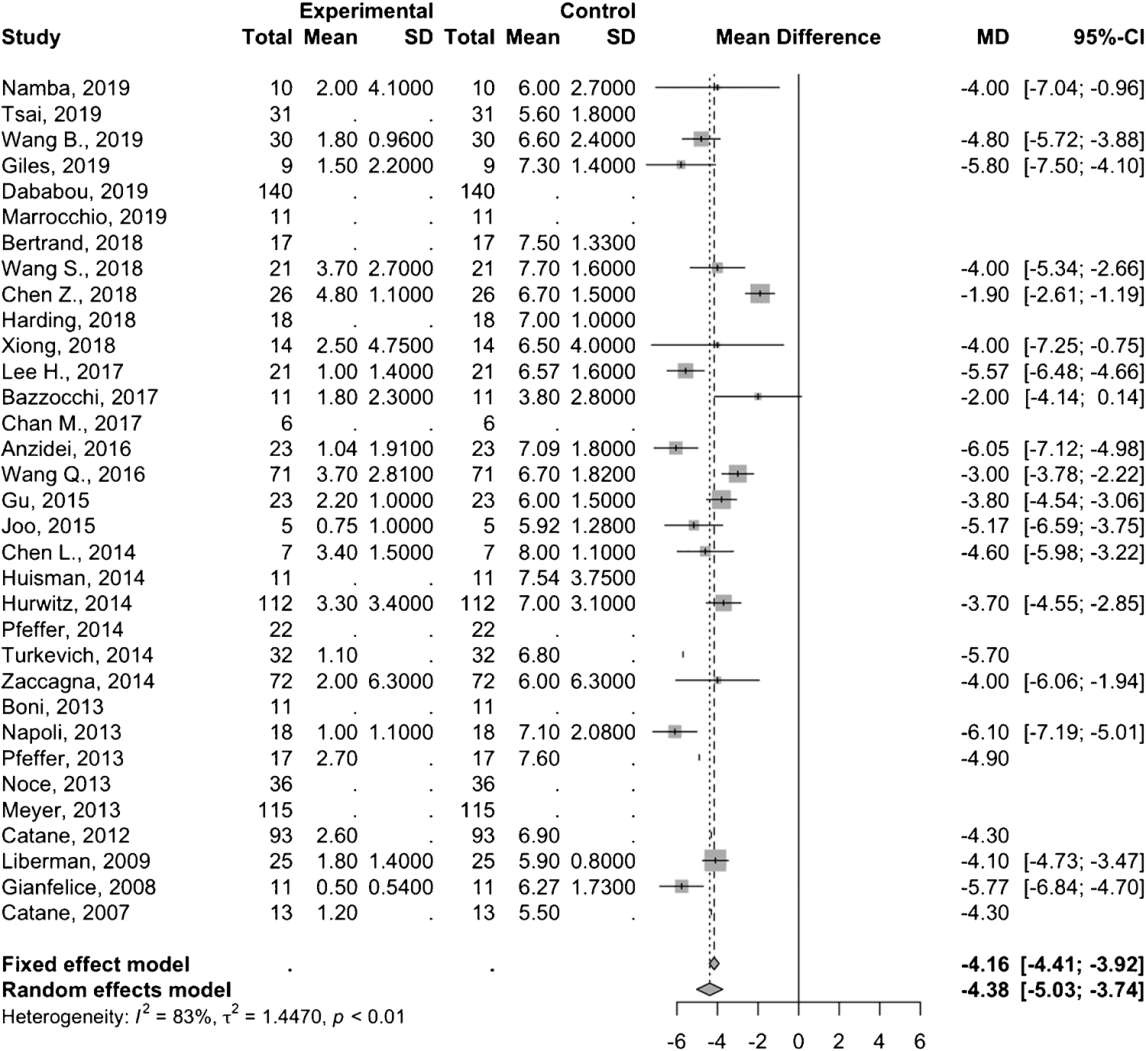
Forest plot of the mean difference in pain scores 3 months after MRgFUS treatment

Twenty studies comprising 543 patients demonstrated as pooled mean difference in pain score of − 3.8 (95% CI − 4.3; − 3.3) and − 4.4 (95% CI − 5.0; − 3.7) at 1-month (Fig. 3) and 3-month (Fig. 4) follow-up, respectively. Of note, one study by Wang et al. [15] involved a pediatric study population and removing this study from the analysis did not significantly change the summary pooled findings (treatment response rate 78% [95% CI 72–83%] with 1-month and 3-month pain score mean differences of − 3.8 [95% CI − 4.3; − 3.4] and − 4.4 [95% CI − 5.0; − 3.7], respectively).

### Prior radiotherapy

In total, 15 studies reported data regarding radiotherapy treatment status prior to MRgFUS treatment. Three studies had patients who had exhausted maximal radiotherapy treatment for their painful bone metastases [17, 20, 30]. There were five studies that reported a fraction of their study population receiving prior radiotherapy. Two clinical trials (*N* = 94) comparing MRgFUS and radiotherapy for painful bone metastases reported that all of their patients in the MRgFUS arm did not receive prior radiotherapy for at least 2–3 weeks before treatment [26, 32]. Five studies (*N* = 112) reported that all of their patients were radiation-naïve prior to MRgFUS treatment. However, the majority (18 studies; *N* = 970) did not provide any information regarding prior radiotherapy treatments. Studies with patients with and without prior radiotherapy achieved treatment response rates of 73.9% and 88.0%, respectively. In one study, Pfeffer et al. demonstrated treatment response rates of 69.0% and 87.5% in patients with and without prior radiotherapy, respectively [32].

### Adverse events

In total, 799 patients across 26 studies were identified to evaluate the rate of MRgFUS-related adverse events in the treatment of painful bone metastases. Among these studies, only seven (0.9%) high-grade toxicity (CTCAE grade ≥ 3) were noted. In detail, high-grade adverse events comprised one DVT, two cases of grade III skin burn, and four fractures. Conversely, 47 (5.9%) low-grade toxicity (CTCAE grade < 3) were recorded. The low-grade adverse events encompassed 24 cases of post-treatment pain, five cases of low-grade skin burns, seven cases of focal edema, eight cases of focal numbness, and three cases of post-treatment fever. Removing Wang et al.’s study [15] involving a pediatric study population did not significantly change the rates of adverse events (high-grade toxicity 0.9%; low-grade toxicity 5.1%). Approximately 94 (11.8%) patients experienced sonication-related pain during MRgFUS treatment.

### Quality of life

Only five studies (*N* = 102) reported data on patient quality of life before and after MRgFUS treatment. Three studies (*N* = 58) utilized the Brief Pain Inventory Quality of Life (BPI QoL) questionnaire [50] with a mean baseline and 3-month follow-up scores of 36.2 and 28.5, respectively [13, 17, 22]. The pooled mean difference was − 8.5 (95% CI − 14.6; − 2.4). Three studies (*N* = 67) used the Quality of Life Questionnaire for patients with bone metastases (QLQ-BM22) [51]. The QLQ-BM22 is subdivided into four subscales to measure distress from pain symptoms (pain site, pain characteristics), where a higher score is indicative of greater distress, and functional status (functional interference, psychosocial aspects), where a higher score indicates greater functional ability [52]. The pooled mean difference from baseline to follow-up scores in the pain site, pain characteristics, functional interference, and psychosocial aspect subscales was − 2.5 (95% CI − 4.6; − 0.41), − 15.8 (95% CI − 21.1; − 10.6), − 12.3 (95% CI − 31.9; 7.4), and − 4.32 (95% CI − 26.2; 17.54), respectively.

Five studies provided changes in pain medication intake in patients following the treatment [15, 31, 34, 35, 39]. Of the patients with reported medication data (unspecified number of patients), an average of 55.8% (range 27.0–76.9%) and 33.0% (range 17.0–67.0%) of patients were able to discontinue or reduce pain medication use after MRgFUS treatment, respectively.

## Discussion

To the best of our knowledge, this is the first meta-analysis on the treatment efficacy and associated toxicity of MRgFUS for painful bone metastases. A systematic review of the current literature identified 33 studies encompassing 1082 patients with painful bone metastases that underwent palliative MRgFUS treatment. Our study showed that MRgFUS is a highly efficacious therapy that is able to provide either complete pain relief or significant pain reduction after 3-month follow-up in the majority of treated patients with symptomatic bone metastases. While only reported in a minority of the selected studies, MRgFUS treatment resulted in the cessation or reduction of pain medication intake in select patients along with improvements in quality of life assessment scores. Moreover, our study exhibited a favorable safety profile of MRgFUS, with a less than 1% rate of severe treatment-related adverse events in the pooled study population and 5.9% rate for low-grade adverse events.

External beam radiation therapy (EBRT) is the current standard local therapy for painful bone metastases and can achieve pain reduction in approximately 60–70% of treated patients [9]. The remaining 30–40% patients not responsive to EBRT are either re-irradiated or undergo alternative treatments, as can be seen in several studies in this meta-analysis [17, 20, 30]. Interestingly, patients that were radiation-naïve experienced higher rates of pain relief compared to patients with prior radiation treatments. Of those who underwent prior EBRT, 74% were able to achieve treatment response, which is higher than the reported 58% response from a prior meta-analysis on re-irradiation therapy for painful bone metastases [53]. This pattern may suggest the presence of radio-resistant bone metastases, which have been predominantly from melanomas, colorectal, or renal cell carcinomas [54]. Our study also suggests an overall treatment efficacy for MRgFUS comparable to radiation therapy. Of note, MRgFUS can be safely repeated for treatment non-responders, since there is no upper limit for the accrued acoustic energy as opposed to the limits in normal tissue tolerance for repeated irradiations from EBRT [55]. Based on the current data, MRgFUS could be considered as an alternative option for painful bone metastases from known radio-resistant cancers.

The mechanism of bone pain relief from MRgFUS treatment is thought to be from thermal denervation of periosteum resulting from the absorption of high acoustic ultrasound energy within cortical bone [39, 56]. Compared to soft tissue tumors, lower energy levels can be used for bone lesions to achieve effective thermal ablation, given the high acoustic absorption rate and low thermal conductivity of cortical bone [57]. As a result, the extent of cortical bone involvement of the bone metastases may indicate likelihood of subsequent treatment response; one study found that lesions with complete cortical destruction were not able to reach the temperature threshold for thermal ablation (> 55 °C) and these patients were unable to achieve significant pain reduction [30]. Other proposed mechanisms include the reduction of mass effect on surrounding tissue from the ablated tumor and the decrease in circulating immunosuppressive cytokines [38, 39, 58]. The high treatment efficacy seen in the current meta-analysis may be partly explained by the ability of MRgFUS to accurately delineate targets from surrounding anatomy with MR imaging, permitting for more precise ablations. MRgFUS also employs real-time thermal mapping and closed-loop control of energy deposition that allows for close monitoring of tissue thermal ablation with complete ablation of gross tumor volume with selected tumor margins [57]. Another notable finding from the current study is the increased mean difference in the pain reduction scores at the 3-month compared to the 1-month follow-up. Several studies have reported new bone formation at the ablation site with progressive formation with subsequent follow-up, which may help mitigate the odds of recurrent pain [35, 39, 40]. Moreover, thermal ablation of bone by MRgFUS has been postulated to stimulate accelerated bone healing with subsequent sclerosis at the ablation site [28].

The image guidance and temperature monitoring that allows for accurate target localization and precise delivery to the target zone may partly explain the low rates of adverse events related to MRgFUS. MRgFUS is a completely non-invasive technique, whereby a high-energy acoustic beam penetrates the body while avoiding significant energy absorption until the target is reached, avoiding damage of surrounding tissue. The most frequent minor adverse effect found in this review was treatment-related pain, followed by low-grade skin burn or edema overlying the treatment site. Pain during treatment can occur if the patient is positioned in a way that applies pressure on the painful body site, which may be the case in older MRgFUS systems where the transducers are in a fixed position [40]. Newer flexible transducer systems can allow for optimal positioning for the patient while maintaining comfort. The severe adverse event rate was only 0.9% (one deep vein thrombosis, two grade III skin burns, and four fractures), which compare well with adverse event rates seen in radiotherapy [59]. In a systematic review, palliative radiotherapy for painful bone metastases was found to have approximately 3% pathologic fracture rate. Fracture risk assessment (e.g., Mirels’ classification) should be performed prior to palliative MRgFUS or radiotherapy to minimize the risk of subsequent pathologic fracture [60]. Lower grade adverse effects associated with radiotherapy include gastrointestinal disturbances, skin reactions, fatigue, and acute pain flare, which seem to depend on the total radiation dose delivered to adjacent normal tissue [61]. Specifically, post-radiotherapy acute pain flare is reported to have an incidence ranging from 40% between 68% compared to only 3% observed with MRgFUS in this study [62]. Depending on lesion location within the bone, different ablation approaches may be used. There are three main energy deposition approaches (near-field approach, direct-treatment approach, soft tissue approach) that can be used for bone metastases, but additional studies comparing these approaches are needed to determine the rates and different types of associated adverse events [63].

Pooled data on quality of life scores indicated a significant decrease in pain or symptom scale from baseline to 3-month follow-up after MRgFUS. This was a consistent finding across two different quality of life questionnaires that were used (BPI QoL and QLQ-BM22). However, the pooled data of the functional scale from the QLQ-BM22 showed non-significant reduction in functional ability or psychosocial aspects after treatment. Among the studies with QLQ-BM22 data, one study showed improvements in both functional status [15] while two studies showed decline after follow-up [14, 22]. These findings indicate a clear benefit of MRgFUS in terms of symptom control but unclear effects on functional ability. Nonetheless, more studies focusing on quality of life are needed.

There are several limitations to our study. As a meta-analysis, the data is limited by the heterogeneity of the included studies. The pooled analysis combined results with varying study types with variable study populations (varying age, primary cancer, variable morphology of treated bone metastases, etc.), reported data, and treatment details/planning (different MRgFUS ablation systems, ablation approach, etc.). The analysis attempted to address this heterogeneity through pooled estimation using fixed and random-effects models, the latter of which is a more conservative measure of the overall effect. In the assessment of mean difference in mean pain scores, estimate standard deviations for several studies were calculated using a standard formula based on provided confidence intervals, as noted above. Additionally, the estimates of quality of life were limited by the use of different questionnaires, and data was only available from a small fraction of selected studies. More prospective studies of MRgFUS treatment utilizing standardized questionnaires are needed to fully assess the impact on quality of life. Finally, the majority of the included studies had follow-up periods that were limited to 3 months.

In conclusion, our systematic review and meta-analysis demonstrated that MRgFUS has moderate efficacy in pain score reduction of symptomatic bone metastases. Our results show safety and efficacy that are comparable standard radiotherapy treatments for painful bone metastases, based on prior studies. Given the low rates of treatment-related adverse events, MRgFUS may be a viable alternative for the palliative treatment of bone metastases in patients with suspected radio-resistant primary cancers or in patients who can no longer receive radiotherapy due to radiation dose restrictions. MRgFUS also proves to be an effective salvage therapy option for patients that fail initial radiotherapy.

## References

[1] Hashim D, Boffetta P, La Vecchia C (2016). The global decrease in cancer mortality: trends and disparities. Ann Oncol.

[2] Kort EJ, Paneth N, Vande woude GF (2009). The decline in U.S. cancer mortality in people born since 1925. Cancer Res.

[3] Coleman RE (2001). Metastatic bone disease: clinical features, pathophysiology and treatment strategies. Cancer Treat Rev.

[4] Weidle UH, Birzele F, Kollmorgen G, Rüger R (2016). Molecular mechanisms of bone metastasis. Cancer Genomics Proteomics.

[5] Selvaggi G, Scagliotti GV (2005). Management of bone metastases in cancer: a review. Crit Rev Oncol Hematol.

[6] Johnstone C, Lutz ST (2014). External beam radiotherapy and bone metastases. Ann Palliat Med.

[7] Westhoff PG, Verdam MGE, Oort FJ (2016). Course of quality of life after radiation therapy for painful bone metastases: a detailed analysis from the Dutch Bone Metastasis Study. Int J Radiat Oncol Biol Phys.

[8] Lutz S, Berk L, Chang E, Chow E, Hahn C, Hoskin P (2011). Palliative radiotherapy for bone metastases: an ASTRO evidence-based guideline. Int J Radiat Oncol Biol Phys.

[9] Chow E, Hoskin P, Mitera G (2012). Update of the international consensus on palliative radiotherapy endpoints for future clinical trials in bone metastases. Int J Radiat Oncol Biol Phys.

[10] Oh D, Huh SJ (2014). Insufficiency fracture after radiation therapy. Radiat Oncol J.

[11] Pacheco R, Stock H (2013). Effects of radiation on bone. Curr Osteoporos Rep.

[12] Mok F, Li K, Yeung R, et al. ‘Who’, ‘when’ and ‘how’ in re-irradiation of recurrent painful bone metastases. J Bone Oncol. 2013;2(1):33‐37. Published 2013 Feb 1. 10.1016/j.jbo.2012.12.003. 10.1016/j.jbo.2012.12.003 PMC4723347 26909270

[13] Namba H, Kawasaki M, Izumi M, Ushida T, Takemasa R, Ikeuchi M (2019). Effects of MRgFUS treatment on musculoskeletal pain: comparison between bone metastasis and chronic knee/lumbar osteoarthritis. Pain Res Manag.

[14] Tsai YC, Lee HL, Kuo CC (2019). Prognostic and predictive factors for clinical and radiographic responses in patients with painful bone metastasis treated with magnetic resonance-guided focused ultrasound surgery. Int J Hyperthermia.

[15] Wang B, Li J, Wei X (2019). Short-term efficacy and safety of MR-guided focused ultrasound surgery for analgesia in children with metastatic bone tumors. Oncol Lett.

[16] Giles SL, Brown MRD, Rivens I (2019). Comparison of imaging changes and pain responses in patients with intra- or extraosseous bone metastases treated palliatively with magnetic resonance-guided high-intensity-focused ultrasound. J Vasc Interv Radiol.

[17] Bertrand AS, Iannessi A, Natale R (2018). Focused ultrasound for the treatment of bone metastases: effectiveness and feasibility. J Ther Ultrasound.

[18] Wang S, Sun Z, Xin C, Du C, Xu L, Gu Y, Li W, Peng W (2018). Magnetic resonance-guided focused ultrasound surgery for pain palliation of bone metastases: Preliminary study on the short-term efficacy and safety. Journal of Interventional Radiology (China).

[19] Chen ZQ, Wang CR, Ma XJ (2018). Evaluation of quality of life using EORTC QLQ-BM22 in patients with bone metastases after treatment with magnetic resonance guided focused ultrasound. Orthop Surg.

[20] Harding D, Giles SL, Brown MRD (2018). Evaluation of quality of life outcomes following palliative treatment of bone metastases with magnetic resonance-guided high intensity focused ultrasound: an international multicentre study. Clin Oncol (R Coll Radiol).

[21] Lee HL, Kuo CC, Tsai JT, Chen CY, Wu MH, Chiou JF (2017). Magnetic resonance-guided focused ultrasound versus conventional radiation therapy for painful bone metastasis: a matched-pair study. J Bone Joint Surg Am.

[22] Xiong H, Zhou Q, Zhang J, Chen Y, Li Q, Tang Y (2018). The safety and short-term efficacy of MR guided focused ultrasound surgery for bone metastases-induced pain palliation. J Therapeutic Ultrasound.

[23] Bazzochi A, Mercatelli D, Facchini G, Guerri S, Gasperini C (2017). Single bone metastasis: role of MR-imaging-guided high-intensity focused ultrasound. Skelet Radiol.

[24] Chan M, Dennis K, Huang Y (2017). Magnetic resonance-guided high-intensity-focused ultrasound for palliation of painful skeletal metastases: a pilot study. Technol Cancer Res Treat.

[25] Anzidei M, Napoli A, Sacconi B (2016). Magnetic resonance-guided focused ultrasound for the treatment of painful bone metastases: role of apparent diffusion coefficient (ADC) and dynamic contrast enhanced (DCE) MRI in the assessment of clinical outcome. Radiol Med.

[26] Wang Q, Wang H, Wang S, Zhang J (2016). Factors affecting the efficacy of the magnetic resonance guided focused ultrasound ablation for painful bone metastases: results from a multicenter study in China. J Ther Ultrasound.

[27] Gu J, Wang H, Tang N (2015). Magnetic resonance guided focused ultrasound surgery for pain palliation of bone metastases: early experience of clinical application in China. Zhonghua Yi Xue Za Zhi.

[28] Joo B, Park MS, Lee SH (2015). Pain palliation in patients with bone metastases using magnetic resonance-guided focused ultrasound with conformal bone system: a preliminary report. Yonsei Med J.

[29] Chen L, Ma C, Meyer J (2014). Quality assurance for MR guided focused ultrasound treatment of bone metastasis: a clinical experience. Int J Radiat Oncol Biol Phys.

[30] Huisman M, Lam MK, Bartels LW (2014). Feasibility of volumetric MRI-guided high intensity focused ultrasound (MR-HIFU) for painful bone metastases. J Ther Ultrasound.

[31] Hurwitz MD, Ghanouni P, Kanaev SV, et al. Magnetic resonance-guided focused ultrasound for patients with painful bone metastases: phase III trial results. J Natl Cancer Inst. 2014;106(5). 10.1093/jnci/dju082 PMC4112926 24760791

[32] Pfeffer RM, Inbar Y, Iozeffi D, Ghanouni P (2014). A retrospective analysis of palliative MRgFUS treatment bone metastases from renal cell carcinoma (RCC). J Clin Onc.

[33] Turkevich V, Kanaev S, Mishchenko D, Savelyeva V (2014). Experience palliative treatment of painful bone metastases with magnetic resonance guided focused ultrasound. J Vasc Interv Radiol.

[34] Zaccagna F, Giulia B, Bazzocchi A, Spinnato P (2015). Palliative treatment of painful bone metastases with MR imaging–guided focused ultrasound surgery: a two-centre study. J Ther Ultrasound.

[35] Napoli A, Anzidei M, Marincola BC (2013). Primary pain palliation and local tumor control in bone metastases treated with magnetic resonance-guided focused ultrasound. Invest Radiol.

[36] Pfeffer R, Catane R, Meyer J, Hurwitz M, Kanaev S, Turkevich V (2013). Palliative effect of MR guided focused ultrasound (MRgFUS) on patients with bone metastases previously receiving sham treatment. Eur J Cancer.

[37] Meyer J, Pfeffer R, Kanaev S, Iozeffi D, Gianfelice D, et al. MR-guided focused ultrasound for painful bone metastases: safety when combined with chemotherapy. J Ther Ultrasound. 2014; 3(suppl 1).

[38] Catane R, Gianfelice D, Kawasaki M, Iozeffi D, Kanyev S (2012). Pain palliation of bone metastases using magnetic resonance guided focused ultrasound-multi-center multi-trial results. Ann Oncol.

[39] Liberman B, Gianfelice D, Inbar Y (2009). Pain palliation in patients with bone metastases using MR-guided focused ultrasound surgery: a multicenter study. Ann Surg Oncol.

[40] Gianfelice D, Gupta C, Kucharczyk W, Bret P, Havill D, Clemons M (2008). Palliative treatment of painful bone metastases with MR imaging–guided focused ultrasound. Radiology.

[41] Catane R, Beck A, Inbar Y (2007). MR-guided focused ultrasound surgery (MRgFUS) for the palliation of pain in patients with bone metastases–preliminary clinical experience. Ann Oncol.

[42] Dababou S, Napoli A, Marrocchio C, Scipione R, Alfieri G, Fierro D, Catalano C (2019). MR-guided focused ultrasound (MRgFUS) versus external beam radiation therapy (EBRT) for the treatment of painful bone metastases: a multicenter, phase III, randomized case-control trial. Cardio Vasc Interv Radiol.

[43] Marrocchio C, Napoli A, Scipione R, Dababou S, Erasmus HP, Catalano C (2019). Magnetic resonance-guided high intensity focused ultrasound (MRgFUS) for the treatment of oligometastatic prostate cancer bone metastases: non-invasive use of sound waves can downstage cancer spread. CardioVasc Interv Radiol.

[44] Boni F, Noce V, Napoli A, Di Mare L, Anzidei M, Brachetti G, Catalano C (2013). MR-guided focused ultrasound ablation on bone metastases: role of dynamic contrast-enhanced MRI in the evaluation of treatment response. CardioVasc Interv Radiol.

[45] Noce V, Napoli A, Brachetti G, Di Mare L, Boni F, Tombolini V, Catalano C (2013). Palliative treatment of bone metastases: analysis of biological effects of MR-guided focused ultrasound (MRgFUS) versus external beam radiation therapy (EBRT) in a randomized comparative trial using functional diffusion maps as an indicator of molecular activity. CardioVasc Interv Radiol.

[46] Liberati A, Altman DG, Tetzlaff J, et al. The PRISMA statement for reporting systematic reviews and meta-analyses of studies that evaluate health care interventions: explanation and elaboration. In: Journal of clinical epidemiology. 2009. 10.1016/j.jclinepi.2009.06.006 19631507

[47] National Institute of Cancer. Common Terminology Criteria for Adverse Events (CTCAE), Version 4.0, DCTD, CTI, NIH, DHHS. 2009.

[48] Higgins JPT, Green S. (editors). Cochrane handbook for systematic reviews of interventions Version 5.1.0 [updated March 2011]. The Cochrane Collaboration, 2011. Available from www.handbook.cochrane.org.

[49] Team RC. R: a language and environment for statistical computing. R Found Stat Comput. 2016.

[50] Kumar SP (2011). Utilization of brief pain inventory as an assessment tool for pain in patients with cancer: a focused review. Indian J Palliat Care.

[51] Zeng L, Chow E, Bedard G (2012). Quality of life after palliative radiation therapy for patients with painful bone metastases: results of an international study validating the EORTC QLQ-BM22. Int J Radiat Oncol Biol Phys.

[52] Chow E, Hird A, Velikova G (2009). The European Organisation for Research and Treatment of Cancer Quality of Life Questionnaire for patients with bone metastases: the EORTC QLQ-BM22. Eur J Cancer.

[53] Huisman M, van den Bosch MA, Wijlemans JW, van Vulpen M, van der Linden YM, Verkooijen HM (2012). Effectiveness of reirradiation for painful bone metastases: a systematic review and meta-analysis. Int J Radiat Oncol Biol Phys.

[54] Rades D, Freundt K, Meyners T (2011). Dose escalation for metastatic spinal cord compression in patients with relatively radioresistant tumors. Int J Radiat Oncol Biol Phys.

[55] Goetz MP, Callstrom MR, Charboneau JW, Farrell MA, Maus TP, Welch TJ (2004). Percutaneous image-guided radiofrequency ablation of painful metastases involving bone: a multicenter study. J Clin Oncol.

[56] Mercadante S, Fulfaro F (2007). Management of painful bone metastases. Curr Opin Oncol.

[57] Jolesz FA (2009). MRI-guided focused ultrasound surgery. Annu Rev Med.

[58] Zhou Q, Zhu XQ, Zhang J (2008). Changes in circulating immunosuppressive cytokine levels of cancer patients after high intensity focused ultrasound treatment. Ultrasound Med Biol.

[59] Bhattacharya IS, Hoskin PJ (2015). Stereotactic body radiotherapy for spinal and bone metastases. Clin Oncol (R Coll Radiol).

[60] Jawad MU, Scully SP (2010). In brief: classifications in brief: Mirels’ classification: metastatic disease in long bones and impending pathologic fracture. Clin Orthop Relat Res.

[61] Chow E, Harris K, Fan G, Tsao M, Sze WM (2007). Palliative radiotherapy trials for bone metastases: a systematic review. J Clin Oncol.

[62] McDonald R, Chow E, Rowbottom L, DeAngelis C, Soliman H (2014). Incidence of pain flare in radiation treatment of bone metastases: a literature review. J Bone Oncol.

[63] Kopelman D, Inbar Y, Hanannel A (2008). Magnetic resonance guided focused ultrasound surgery. Ablation of soft tissue at bone-muscle interface in a porcine model. Eur J Clin Invest.

